# Supplementary material for: Are carbon emissions trading and green financial instruments synergistic? -Comprehensive quantitative research based on content analysis
Source: PLoS One. 2024 Mar 7;19(3):e0298601. doi: 10.1371/journal.pone.0298601 (PMC10919601; doi:10.1371/journal.pone.0298601)
Supplement: S1 Data — (ZIP) [file pone.0298601.s001.zip › Data/地级市Z_F工作报告.zip!/╣π╬≈/╬α╓▌╩╨/└┤╫╘╣1⁄2╓┌║┼í╛┬φ┐╦╩2╛▌═°í┐_2011.docx]

梧州发布微信

智能问答

移动版

网站支持IPv6

各位代表：

　　现在，我代表市十二届人民政府，向大会作政府工作报告，请予审议，并请市政协委员和其他列席人员提出意见。

过去五年的工作

过去的五年，是我市改革发展进程中极其重要的五年。面对国际金融危机的严重冲击和各种自然灾害的严峻考验，我们在自治区党委、政府和市委的正确领导下，紧紧依靠和团结带领全市人民，围绕区域性枢纽城市、西江黄金水道中心城市和承接产业转移示范基地建设，抢抓机遇，克难攻坚，开拓创新，实现了交通由广西末梢向国内区域性综合交通运输枢纽转变，工业由工业化初期向中期转变，经济由起步阶段向快速成长阶段转变，实现了地区生产总值、财政收入、全社会固定资产投资等15项指标翻番，开创了跨越式发展的崭新局面。

过去的五年，是综合实力显著增强的五年。全市地区生产总值由2006年的270.4亿元增加到2010年的579.3亿元，年均增长16.1%，人均生产总值达到19430元；财政收入由23.1亿元增加到56.1亿元，年均增长22.7%；全社会固定资产投资由121.9亿元增加到468.4亿元，年均增长35.8%，累计完成1268亿元，是“十五”时期的4.77倍。县域经济不断壮大，岑溪市三次、苍梧县和藤县两次进入广西科学发展十佳县行列，蒙山县两次荣获广西科学发展进步奖。

过去的五年，是经济结构不断优化的五年。三次产业比例为9.5:62.9:27.6，二、三产业占GDP的比重为90.5%，比“十五”末提高12.3个百分点。工业企业规模不断扩大，全市大中型企业发展到42家，规模以上工业企业近500家，年销售收入超亿元企业270家，年纳税超亿元企业6家，上市公司3家。工业企业效益稳步提升，市本级40家重点企业产值、盈亏相抵后实现利润、税收入库均实现大幅增长。工业支撑带动作用显著增强，全市工业总产值由2006年的330.6亿元增加到2010年的868.9亿元，年均增长26.3%；工业化率由1.6提高到4.2，工业对经济增长的贡献率达到71.9%。园区经济快速发展，2010年“五园八区”实现工业总产值270亿元，占全市工业总产值的31.1%。

过去的五年，是农业基础明显巩固的五年。全市农业总产值由2006年的91.1亿元增加到2010年的129.8亿元，年均增长5.1%。粮食种植面积保持在230万亩以上，粮食年产量保持在80万吨以上。特色农业稳步发展，形成桑蚕、名优水果、六堡茶、三黄鸡、江河网箱养鱼等十大优势产业和特色农产品生产基地，建成广西最大的砂糖桔生产基地，六堡茶获得国家地理标志产品保护。农业产业化步伐加快，全市拥有国家级、自治区级和市级农业产业化重点龙头企业32家，广西华虹蚕丝股份有限公司、梧州松脂股份有限公司进入国家级农业产业化重点龙头企业行列。农田水利建设力度加大，病险水库除险加固、堤防加固、农田灌溉等工程建设累计完成投资17.88亿元。建成社会主义新农村示范点19个、生态富民示范村21个。

过去的五年，是交通枢纽地位凸显的五年。洛湛铁路建成通车，南广铁路梧州段加快建设，岑溪至罗定铁路、梧州港赤水圩作业区进港铁路开工建设。梧州至南宁、梧州至广州、梧州至桂林、岑溪至筋竹高速公路建成通车，梧州至贵港、岑溪至水汶、市区环城高速公路开工建设，形成梧州到主要出行目的地的“3小时经济圈”。县县通二级以上公路，乡镇通硬化路，基本实现村村通公路。梧州至南宁、重庆、贵阳、珠海航线开通。西江亿吨黄金水道建设加快推进，梧州至贵港Ⅱ级航道建成，长洲水利枢纽投产、三线四线船闸开工建设，李家庄码头一二期扩建工程竣工，梧州港赤水圩作业区一期工程投入运营。2010年全市港口货物吞吐量达到1600万吨，集装箱吞吐量达20万标准箱。国内区域性综合交通运输枢纽地位基本确立。

过去的五年，是城市面貌极大改观的五年。旧城改造力度加大，先后投入近8亿元实施平民冲、冰泉冲、石鼓冲地质灾害综合整治和城市棚户区改造，帮助近5000户居民搬离地质灾害高危地区。红岭新区路网框架基本形成，已开工建设道路20条、全线贯通12条。南岸片区开发稳步推进，苍海项目开工建设。城市道路、桥梁、供水、供气、公共交通等设施进一步完善，城市综合承载能力明显提升。积极实施城乡风貌改造、城乡清洁和“穿衣戴帽”工程，完成一批城市沿街立面和城市景观改造项目，城市风貌特色鲜明，城市环境生态宜居。城镇化进程加快，城镇化率由2006年的32.6%提高到46%，高于全区平均水平。

过去的五年，是改革开放纵深推进的五年。国有企业、财税、金融、投融资等重点领域改革和医药卫生体制改革、农村综合改革取得积极进展，行政管理机构新一轮改革任务全面完成。对外贸易稳步发展，累计实现进出口总额26.65亿美元，年均增长10.8%。积极参与中国-东盟自贸区、泛珠三角、泛北部湾等多区域合作，承接产业转移成效显著，共引进项目1856个，实际到位资金458.5亿元。我市被确定为国家西部大开发广西桂东承接产业转移示范区和国家加工贸易梯度转移重点承接地。成功举办了五届梧州国际宝石节、三届西江经济发展论坛，城市影响力、竞争力不断提高。

过去的五年，是生态建设持续加强的五年。万元生产总值能耗下降到1.054吨标准煤，二氧化硫和化学需氧量排放总量控制在自治区下达的指标之内，超额完成“十一五”节能减排目标任务。全面推进环保基础设施建设，建成12座污水和生活垃圾处理设施，城镇污水集中处理率和生活垃圾无害化处理率分别达到83%和100%。积极利用再生资源发展循环经济，梧州再生资源循环利用示范园区被确定为国家循环经济———“城市矿产”示范基地。强化生态环境保护，江河水质达标率、省界界首江河断面水质达标率、空气质量优良率均保持100%。全市森林覆盖率达74.8%，荣膺广西首个“国家森林城市”。

过去的五年，是社会事业全面进步的五年。科技创新能力不断增强，组建自治区级千亿元产业研发中心2个、企业技术中心14个、工程技术研究中心4个。公共检验能力和科研水平进一步提升，建设国家级和自治区级检验中心4个。教育事业协调发展，梧州学院“申本”成功，普高教育质量位居全区前列，职业教育资源整合加快推进，职业教育攻坚通过评估验收，农村教育“两基”攻坚目标和城乡免费义务教育全面实现。社会保险覆盖面不断扩大，保险待遇大幅提高，各类保险的平均参保率达到90%。公共卫生和基本医疗服务体系不断完善，新型农村合作医疗参合率达90%以上。城乡居民最低生活保障标准逐年提高。公共文化服务体系逐步完善，新建16个乡镇文化站、39个村级公共服务中心、211家农家书屋。加强城乡社区体育设施建设，全民健身活动深入开展。积极推进旅游目的地和游客集散地建设，骑楼城、石表山被评为国家4A级旅游景区。人口和计划生育管理服务水平进一步提高，人口自然增长率控制在10％。以内，我市获得自治区人口和计划生育工作进步奖，蒙山县获“全国计划生育优质服务县”称号。

过去的五年，是群众得到实惠最多的五年。坚持为民办实事，在就业、社会保障、“三农”、教育、医疗、卫生等民生领域累计投入资金164.5亿元，人民群众生活水平和质量进一步提高。2010年全市城镇居民人均可支配收入16427元，年均增长15.1%；农民人均纯收入4879元，年均增长13.8%，高于全区平均水平；社会消费品零售总额191.8亿元，年均增长17.5%。实施灵活多样的就业方式，新增城镇就业10.87万人，转移农村劳动力28.42万人，城镇登记失业率控制在4.5%以内。大力实施安居工程，改造农村危旧房5360户、城市棚户区3500户，建成廉租房、公租房、经济适用房、限价房等保障性住房8754套，新开工建设各类保障性住房13140套。

过去的五年，是社会大局和谐稳定的五年。积极开展“平安梧州”创建活动，强化对矛盾纠纷的排查调处，及时解决了一批涉及群众切身利益的热点难点问题。深入开展食品药品安全、安全生产专项整治活动，安全形势总体保持稳定。组建了广西第一支综合应急专业救援队伍，突发公共事件应急处置能力进一步提高。战胜了历史罕见的雨雪冰冻、干旱、严重洪涝和特大地质灾害，应急抢险救灾和恢复重建工作有力有序有效，建成灾民新村18个，帮助11943户因灾倒房户住进新居。推进社会治安防控体系建设，成立了广西第一支特巡警支队，建成“天网工程”，有效预防和打击了各种违法犯罪活动。圆满完成“五五”普法任务。强化社会治安综合治理，加强重要节日和重大活动期间的稳定工作，切实维护社会和谐稳定，连续五年被自治区授予“平安市”称号。

五年来，政府自身建设进一步加强。行政审批制度改革不断深化，行政审批事项不断精简压缩。依法行政能力和水平进一步提升，法制环境明显改善，机关绩效考评工作扎实推进。政务公开力度加大，电子政务建设加快。深入开展作风效能建设，建立了市、县两级政务服务中心和行政效能电子监察系统，政府机关办事效率和服务水平明显提高。切实加强工程建设领域突出问题、商业贿赂和小金库等专项治理，严肃查处各类违法违纪案件，及时纠正损害群众利益的不正之风，有效维护了廉洁高效的政府形象。

五年来，我们自觉接受人大及其常委会的法律监督、工作监督和政协的民主监督，认真办理人大代表议案、建议以及政协提案，办结率保持100%，市政府被评为自治区政协提案承办先进单位。国防动员和民兵预备役工作扎实推进，双拥工作深入开展，军政军民更加团结。驻梧部队在支持地方建设、处置突发事件等方面作出了重要贡献。工商、税务、金融、海关、检验检疫等中直、区直驻梧单位为经济社会发展提供了强有力的支持。工会、共青团、妇联等组织在促进经济社会发展中发挥了积极作用。民族、宗教、外事、侨务、对台、人防、统计、供销、扶贫、水库移民、农机、广播电视、社会科学、市志、档案、妇女儿童、老龄、残疾人等工作都取得了新成绩。

今年是“十二五”的开局之年，我们在银根从紧、地根从严、物价高企、煤电紧张等复杂严峻的形势下，扎实推进各项工作，继续保持了经济较快发展的良好势头。1至8月，预计全市完成地区生产总值420亿元，同比增长15%；规模以上工业总产值587亿元，增长49.8%；财政收入47.5亿元，增长34.7%；全社会固定资产投资433.2亿元，增长34.3%；社会消费品零售总额136亿元，增长16.8%；城镇居民人均可支配收入12320元，增长12%；农民人均现金收入3960元，增长15%。

五年的实践充分证明，推动梧州经济社会又好又快发展，最根本的就是要以科学发展观统领经济社会发展全局，最重要的就是要保持社会大局的和谐稳定，最基本的就是要：坚持解放思想。没有思想的领先，就没有发展的领先；实现不了观念的超越，就实现不了发展的超越。只有思想上的大解放，才能推动梧州的大发展。坚持科学发展。只有把握发展规律、创新发展理念、转变发展方式、破解发展难题，才能做到好中求快、稳中求进、危中求机、紧中求活，提高发展质量和效益，不断增强发展的协调性，走出一条生产发展、生活富裕、生态良好的科学发展道路。坚持投资拉动。梧州作为后发展、欠发达地区，投资拉动仍是最直接、最有力、最有效的途径，只有坚定不移地抓投资上项目，保持经济长期平稳较快发展，才能不断壮大经济总量。坚持“三换”战略。只有科学审视市情，抢抓多区域合作的新兴机遇，以空间换时间，以资源换产业，以存量换增量，才能在开放合作中承接产业转移、聚集生产要素、优化产业结构、推动产业振兴。坚持“三力”建设。只有不断强化执行力、协同力、创新力，才能在工作中做到贯彻有力、“干”字当头，互相支持、协同推进，用新观念、新思维、新视角、新举措，推动经济社会跨越发展。坚持民生为重。只有始终把人民放在心中最高位置，做到发展依靠人民，发展为了人民，发展成果由人民共享，着力提高人民生活水平，着力提高人民幸福感，才能形成共建共享和谐社会的生动局面。

各位代表，回顾这浓墨重彩的五年，我们为梧州发生的巨大变化而骄傲，为全市人民创造的辉煌业绩而自豪！这是自治区党委、政府和市委坚强领导的结果，是市人大、市政协和社会各界监督支持的结果，是全市人民群众团结奋斗、顽强拼搏的结果。在此，我代表市人民政府，向全市各族人民，向人大代表、政协委员，向各民主党派、工商联、无党派人士，向中直、区直驻梧单位，向驻梧部队官兵和政法干警，向所有关心支持梧州发展的社会各界人士，致以崇高的敬意和衷心的感谢！

在肯定成绩的同时，我们也清醒地认识到，我市后发展、欠发达的基本市情尚未改变，经济社会发展中还存在不少突出矛盾和问题。主要表现在：经济总量不大，人均水平较低，综合实力与发达地区相比仍有较大差距；产业结构不合理，科技创新能力不强，区位优势、资源优势和交通优势尚未很好地转化为经济优势、竞争优势，支撑跨越发展的基础还不牢固；社会事业发展相对滞后，公共服务保障能力有待提高；政府自身建设有待进一步加强等。对此，我们一定高度重视，认真加以解决，决不辜负人民群众的期望。

今后五年的奋斗目标

各位代表，今后五年，是我市科学发展、和谐发展、跨越发展的关键期，是深化改革开放、加快转变经济发展方式的攻坚期。我们要牢固树立责任意识、机遇意识和忧患意识，凝聚全市各族人民的力量，充分利用各种有利条件，努力实现经济社会又好又快发展。

根据市第十二次党代会精神和市“十二五”规划纲要，今后五年政府工作的总体要求是：高举中国特色社会主义伟大旗帜，以邓小平理论和“三个代表”重要思想为指导，深入贯彻落实科学发展观，以科学发展为主题，以加快转变经济发展方式为主线，紧紧抓住新一轮西部大开发、西江经济带建设等重要战略机遇，围绕实现富民强梧新跨越，加快对接珠三角，主动融入北部湾，夯实农业和交通基础，走好经济发展“三步棋”，唱响城市建设“三部曲”，着力打造“一带两地一枢纽一园区”，加快建设“两城市一基地”，使经济发展更好更快，城市发展更大更美，人民生活更加富裕更有尊严，为全面建设小康社会打下具有决定性意义的基础。

面对新形势、新任务和新要求，我们必须自我加压，奋力前行。今后五年的奋斗目标是：

———城市聚集力显著提升。到2015年，地区生产总值超1000亿元，年均增长12% 以上；工业总产值超2000亿元，年均增长18%以上；固定资产投资超940亿元，年均增长15%以上；财政收入突破120亿元，年均增长15%以上；城市人口达100万人，城市建成区面积达100平方公里。

———枢纽地位显著提升。基本建成以西江航运干线、“一环七射三连线”高速公路网、“丰字型”铁路枢纽、梧州机场航空港及西气东输二线为骨架，水路、公路、铁路、空港和管道运输五位一体的国内区域性综合交通运输枢纽。到2015年，铁路运营里程达到398公里，公路通车里程达到6100公里（其中高速公路482公里），港口吞吐能力达到5000万吨以上，按干线机场标准完成机场迁建。

———开放层次显著提升。全面实施“东向”战略，努力把梧州建设成为粤桂合作的先行区、国家东西部融合发展的试验区、西江经济带的核心区。西部大开发广西桂东承接产业转移示范区和国家加工贸易梯度转移重点承接地建设取得重大进展，旅游目的地和游客集散地建设初具规模。

———生态质量显著提升。加强生态建设，优化人居环境，实现人与自然和谐发展。到2015年，单位GDP能耗和二氧化碳排放量进一步下降，主要污染物排放总量达到控制目标，工业固体废弃物处理率达到90%，重点工业企业废水排放达标率100%，主要江河水质保持国家Ⅲ类标准以上， 市区及各县（市）城区的城镇集中式饮用水源地水质达标率100%，空气环境质量优良率保持100%。全市森林覆盖率高于75%，城市建成区绿化覆盖率40%以上，成为国家园林城市。

———生活水平显著提升。城乡居民收入与经济发展同步，劳动报酬与劳动生产力提高同步。到2015年，城镇居民人均可支配收入达29000元，农民人均纯收入达9000元；社会就业更加充分，社会保障更加有力，公共服务更加健全，文化软实力进一步增强，基本实现学有所教、劳有所得、病有所医、老有所养、住有所居、困有所济，社会更加和谐、人民更加幸福。

我们相信，再经过五年的努力，一个经济日益发达、文化日益繁荣、社会日益和谐、生态日益文明、人民日益富裕的新梧州，必将呈现在世人面前！一个更有实力、更具活力、更富魅力的新梧州，必将焕发出更加璀璨夺目的光彩！

今后五年的主要工作

实现以上奋斗目标，今后五年要着重抓好以下工作：

一、 做强产业

培育壮大工业产业。优先发展再生资源循环经济产业集群，重点发展陶瓷、机械制造、医药、食品和光电信息等产业集群。加快打造千亿元、百亿元产业园区，到2015年梧州再生资源循环利用示范园区建成千亿元产业园区，陶瓷产业园区、不锈钢产业园区和梧州工业园区建成300亿元产业园区，全市园区总产值占全部工业总产值的比重达70%以上。打造西江经济带核心区，到2015年核心区工业总产值达1700亿元，工业增加值超500亿元。加快实施“抓大壮小扶微”工程，到2015年培育年产值达100亿元的企业1家以上，50至100亿元的企业2家以上，20至50亿元的企业10家，10至20亿元的企业20家，新增成长性好的中小企业500家，力争上市公司10家。积极发展电子信息、生命健康、新材料、新能源等战略性新兴产业，不断形成新的经济增长点。突出抓好以掌上多媒体为主的电子信息产业，力争引进10至20家品牌优质企业，努力建设区域性移动互联网终端产品制造基地。

提升农业产业化水平。依法保护耕地，稳定粮食生产，确保粮食年总产量达85万吨以上。加快转变农业发展方式，大力发展高产、优质、高效、安全的生态特色农业，全面提高农业综合效益，重点推进西江经济带梧州特色农业示范区建设。优先发展瘦肉型猪、三黄鸡、肉兔、网箱鱼、蜂蜜等特色养殖业，推进优质畜禽水产品养殖规模化，力争到2015年畜牧水产业占农业总产值比重50%以上。发展壮大六堡茶、砂糖桔、中草药等特色优势种植业，努力形成规模化的特色农业产业带。加快特色林业和林下经济发展，重点培育速丰林、高脂松等原料基地，加速形成以林浆纸（纤维）、林板、松香和天然香料香精为主的林业产业集群。加快发展农业产业化重点龙头企业，培育5个国家级、20个自治区级重点龙头企业，力争年产值超5亿元龙头企业达3家以上。到2015年农业总产值达166亿元，年均增长5%以上。

加快发展现代服务业。围绕西江经济区域服务业中心建设，大力发展生产性物流业，积极引进大型商贸物流企业。建设赤水港综合服务物流园区、红岭商贸物流园区和中恒医药、建材、农副产品、日用品4大商贸物流城，以及汽车、钢材、机电、纺织、零部件等30个专业市场。打造河东老城、红岭新区、南广铁路站前广场3个商业圈，建设科学文化艺术、珠宝饰品、滨江休闲饮食、骑楼民俗文化、新加坡印象等5条特色街区。加强“引金入梧”工作，争取引进股份制银行、农村商业银行5家以上，力争设立西江银行。强化投融资平台建设，完善社会信用担保体系。推进数字城市和物联网应用基地建设，构建适应西江经济区域快速发展的信息服务体系。推进旅游目的地和游客集散地的建设，重点打造“10+2”旅游项目，加快形成“一核四组团”旅游景区发展格局，到2015年实现接待游客1300万人次，旅游总收入128亿元。

二、 做牢基础

完善现代综合交通格局。建成南广铁路、贵梧高速公路、柳梧高速公路、市环城高速公路、西气东输二线梧州段，完成梧州机场迁建。建成长洲水利枢纽三四线船闸、梧州港赤水圩作业区码头二期、紫金村码头、李家庄码头三期等一批水运基础设施。争取开工建设柳肇铁路梧州段、柳韶铁路蒙山段、荔浦经蒙山至玉林高速公路、梧州经封开至广佛肇高速公路，开展贵港至梧州3000吨级航道、桂江航道整治。做好洛湛铁路增建二线及电气化改造工程、梧信高速公路、贺州经蒙山至巴马高速公路等项目前期工作。完成龙圩至潭东、三龙至津北等一批路网工程。实现县县通高速、乡镇通油路、行政村通硬化路。加快城乡公共交通发展，改善市区公共交通体系，推进城乡客运一体化。

加强水利基础设施建设。把水利摆在更加突出的位置，以民生水利为重点，提升城乡防洪标准和防灾能力，全面加固城市堤防，建成长洲南北堤、下小河防洪排涝工程，开工建设塘源、高旺和三龙防洪堤。加强气象、水文监测设施建设，提高灾害监测技术水平。抓好9大灌区续建配套与节水改造工程建设。全面完成72座病险水库的除险加固。加快推进农村饮水安全工程，解决60万农村人口特别是农村中小学饮水安全问题。到2015年完成水利投资50亿元，基本建成与经济社会发展相匹配的现代水利基础设施。

强化能源支撑能力。增强电力供应保障能力，重点抓好500千伏、220千伏、110千伏等一批输变电工程的建设。全面改造城乡电网，建设完善的现代化电网。建成旺村水利枢纽，推进小水电站建设。继续推进核电、火电项目前期工作。积极发展生物质能、沼能、太阳能等可再生能源，大力推广天然气综合利用。

　　三、做大城市

加快中心城市建设。全面建设红岭新区，重点推进红岭路网、三龙滨江大道、供水管网等基础设施建设，建成西江三桥、中央商务区、青少年活动中心、玫瑰湖景区等一批重大项目。整体开发南岸片区，建成西江四桥和主要交通网络，基本形成新城区雏形。加快建设苍海城市湿地生态区、八宝塘新城和长洲岛旅游休闲基地，开工建设国际游艇俱乐部和岭南亚太影视城等项目。全面展开市区地质灾害综合整治，加快棚户区和城中村改造。加快县域中心城镇建设，苍梧县建成与中心城区一体化发展的城乡统筹示范城市，岑溪市建成桂东南副中心城市，藤县建成新兴工业化中等城市，蒙山建成桂东度假休闲精品小城市。培育发展建制镇、特色小城镇，推进城镇面貌改观“三年行动计划”，打造一批传统文化突出、综合承载能力较强的重点镇。2015年城镇化率达到52%以上，超过全国平均水平。

推进园林城市、花园城市建设。深入实施城市森林建设中期规划，进一步完善城市绿网、水网、路网。建设一批具有岭南历史文化特质的主题公园、植物园、森林公园、花园街、花园式住宅小区，优化生态宜居环境。力争到2015年，城市建成区绿化覆盖率达40%以上，绿地率达31%以上，人均公园绿地面积达8平方米以上。

提高城市管理服务水平。建设市政公用基础设施信息化管理和公用事业服务系统，提高城市管理信息化水平。深化城乡清洁工程，确保城市环境综合治理全域覆盖、保持常态。加强城市停车场规划建设，形成良好的城市交通秩序。提高重点区域、重点地段和标志性建筑的设计水平，集中打造一批城市亮点和建筑精品，进一步提升城市形象。加强市民素质教育，不断提高城市文明程度，努力创建全国文明城市。

四、 做活机制

深化重点领域改革。加大改革力度，加快破除制约科学发展的体制机制障碍。深化财政体制改革，加快构建公共财政、透明财政和效率财政。创新国有资产管理机制。推进地方金融机构、金融管理体制改革。加快推进新型农村金融机构建设。大力推进科技、教育、文化、卫生等事业单位分类改革。深化土地有偿使用制度改革，推进城乡建设用地增减挂钩工作，保持耕地总量动态平衡。全面完成农村集体林权制度改革。积极推进水、电、气等资源性价格和环境收费改革。

拓展开放合作空间。对接珠三角，推动粤桂合作试验区建设，加快融入广佛肇城市圈，主动接受先进生产力辐射带动。融入北部湾，参与中国-东盟自贸区合作与交流。加强与日本、韩国、新加坡及港澳台地区的经贸合作和文化交流。高水平办好梧州国际宝石节、西江经济发展论坛，进一步加强西江流域城市合作。加快培育出口基地，着力培植一批市场竞争力强的出口龙头企业。加快保税仓和监管区的建设，争取设立梧州出口加工区。开工建设广西电子口岸物流服务平台梧州分平台。支持和鼓励有条件的企业开拓国外市场，促进经济技术合作交流。到2015年，全市进出口总额达到13亿美元，年均增长15%以上。

创新招商引资方式。依托梧州自身优势，大力实施产业招商，引进大项目好项目，形成大配套，发展大产业。坚持大招商招大商招好商，紧盯世界500强、中国500强、区内50强和行业龙头，着力引进投资规模大、技术含量高、带动能力强的大企业、大项目，力争在引进重大项目上有新突破。大力发展民营经济，完善和落实各项优惠政策，鼓励和促进民营资本进入基础设施、公用事业、保障性住房、文教卫体、环境保护等领域。提高利用外资的质量和水平，鼓励外商投资研发中心、高新技术产业、先进制造业和节能环保产业，鼓励外商投资传统产业的技术改造和升级，鼓励外商投资优势资源开发、现代农业、现代服务业。今后五年，招商引资实际到位资金年均增长20%以上。

　　五、做优生态

大力发展循环经济。树立可持续发展的理念，开展循环经济园区和低碳经济园区试点。制定实施不同发展功能区产业准入标准，促进经济发展与环境保护有机结合。加大循环经济投入，促进生产、流通、消费各环节的循环经济发展。加快建立城市垃圾回收体系，科学布局和改造回收网点。逐步推行城市生活小区垃圾分拣，建立完善垃圾分类收集和分选处理系统，实现垃圾无害化、资源化利用。推进生态农业建设，大力发展生态服务业。加快城市供水管网向农村延伸，逐步推进城乡水务一体化，最大限度地提高水资源利用率，实现经济效益、生态效益和社会效益的统一。

建设城市矿产基地。重点建设30万吨再生铜、30万吨再生铝、150万吨再生不锈钢、2万吨铝合金精密压铸件、20万吨无氧铜杆等项目，加快发展再生铜、再生铝、再生不锈钢、再生塑、再生棉产业集群，加快形成回收、拆解、加工、深加工、交易一体化的循环经济产业体系。到2015年循环经济产业实现工业总产值1000亿元以上、增加值300亿元以上。

加大节能减排力度。开展企业节能低碳、绿色建筑和节能减排全民行动。全面推广清洁生产，对污染源工业废水排放实行自动在线监控，确保达标率100%。加快完善工业园区污染治理设施，改造生活垃圾污水处理设施，建成餐厨垃圾处理中心，完成市生活垃圾处理场西区填埋场封场，强化治污设施运行监控监管，实现污染物全面达标排放。建成市医疗废弃物处置中心，全市医疗废弃物无害化处置率达到100%。加强水环境质量和空气质量监管。建设市区饮用水取水和备用水源工程。

加强生态环境保护。积极开展生态市县、生态镇村建设，加强农村环境综合整治。强化对水源、土地、森林、河流等自然资源的保护，促进自然生态系统的良性循环。加快重要生态功能保护区建设，规范自然保护区管理。加强资源开发类建设项目的生态环境保护、恢复和管理，切实推进矿山环境治理和生态恢复，进一步规范稀土开发与合理利用。实施“绿满梧州”工程，打造以沿西江、桂江为重点的绿色长廊。

　　六、做实民生

实施安居乐业工程。把改善居民特别是低收入家庭和困难群体的住房条件作为政府工作的重点，实施“安居”工程，加快保障性住房建设，大力推进城乡危旧房、棚户区改造。用3年时间，建设廉租房和公租房100万平方米、限价房和经济适用房100万平方米，改造城乡危旧房、棚户区100万平方米。实施“乐业”工程，鼓励和促进全民创业，千方百计增加就业岗位。到2015年新增就业30万人以上，城镇登记失业率控制在5%以内。

健全社会保障体系。全面实施医疗保险市级统筹，到2015年实现基本医疗保障制度全面覆盖城乡居民。健全城镇职工和居民养老保险制度，实现新型农村社会养老保险制度全覆盖。推进事业单位养老保险制度改革。完善失业、工伤、生育保险制度。健全城乡居民最低生活保障和失地农民社会保障制度，合理提高低保标准，对符合条件的困难群体实现应保尽保。完善城乡一体化社会救助和福利服务体系。

发展社会各项事业。实施第五轮科技创新计划，开展全民科学素质行动，每万人拥有发明专利量明显增加。优先发展教育，深化教育教学改革，调整优化城乡教育布局，扎实推进学前教育、特殊教育、义务教育、普高教育、职业教育和高等教育协调发展，办人民满意的教育。深入实施人才强市战略，创新人才培养、选拔、激励和评价机制，认真抓好各类人才队伍建设。建立健全医疗卫生服务体系，优化配置医疗卫生资源，形成三级医院2个半小时、二级医院1个半小时和社区卫生服务机构15分钟服务圈，提高人民群众的医疗卫生服务保障水平。实施文化惠民工程，完善基层公共文化设施网络，提升公共文化服务水平。弘扬地方特色文化，繁荣粤剧、牛娘戏等地方剧种。完善体育基础设施建设，深入开展群众性体育活动，不断提高竞技体育水平。全面加强人口和计划生育工作，稳定低生育水平，进一步提高出生人口素质。加强国家安全和青少年思想道德教育。抓好国防动员和民兵预备役工作。培育发展行业商（协）会、同业公会。大力发展残疾人事业、老龄事业和慈善事业。切实加强民族宗教、妇女儿童、保密、档案、法制、人防、地方志等工作。

加强社会管理与创新。加大政府公共服务建设力度，加强城市社区建设和管理，积极推进新型农村社区发展，基本实现城乡社区公共服务中心全覆盖。深入开展“平安梧州”创建活动，构建社会和谐稳定模范城市。全面实施警务机制改革，突出抓好社会治安综合治理，严密防范和依法打击各类违法犯罪行为。以群众工作统揽信访维稳工作，完善“大调解”工作体系，前移社会矛盾化解关口。健全应急管理体系，妥善应对群体事件和突发公共安全事件。实施企业安全标准化管理，强化企业安全生产主体责任，防范和遏制重特大事故发生。加强食品药品监管，保障人民群众饮食和用药安全。千方百计抑制物价过快上涨，切实保障人民群众特别是低收入群体基本生活水平不降低。

　　各位代表！实现富民强梧新跨越，需要全市上下的共同努力，需要政府自身的积极作为。一是要进一步转变政府职能。科学配置政府部门行政管理职能，全面履行经济调节、市场监管、社会管理和公共服务职能，加快实现政企、政资、政事分开和政府与中介组织分开。充分发挥政府和市场"两只手"的作用，善于运用市场机制解决发展中的问题。二是要进一步提高行政效能。深化机关行政效能建设，严格执行“三项制度”。完善市、县两级政务服务中心建设，打造便民、高效的政务服务平台。大力推进政务公开，加强电子政务、政风行风热线和政府网站建设，营造高效运转的政务环境。三是要进一步推进依法行政。自觉接受人大监督和政协民主监督，强化监察、审计等专门监督，高度重视社会公众监督和新闻舆论监督。严格落实行政执法责任制和过错责任追究制，不断完善执法监督约束机制。继续推进科学民主决策，健全社情民意反馈机制，努力提高政府决策的透明度和公众参与度。四是要进一步加强廉政建设。认真落实党风廉政建设责任制，完善惩治和预防腐败体系。加强“三公”经费管理，推进公务用车制度改革，不断降低行政运行成本。积极稳妥推进预算公开，依法理财、民主理财。加强对工程建设、土地使用权出让、矿产资源开发和政府采购等重点领域的监管，坚决查处腐败案件，树立风清气正的良好形象。我们一定勤勉尽责，以勤政造福于民，以优政服务于民，以廉政取信于民。

　　各位代表，实现富民强梧新跨越，是时代的要求，是人民的期盼，是历史赋予我们的神圣使命。让我们紧密团结在以胡锦涛同志为总书记的党中央周围，高举中国特色社会主义伟大旗帜，深入贯彻落实科学发展观，在市委的坚强领导下，解放思想，团结拼搏，锐意进取，扎实工作，为夺取全面建设小康社会新胜利，谱写人民美好生活新篇章而努力奋斗！
